# Associations Between Smoking, Alcohol Consumption, Physical Activity and Depression in Middle-Aged Premenopausal and Postmenopausal Women

**DOI:** 10.3389/fpsyt.2021.761761

**Published:** 2021-12-23

**Authors:** Hyewon Kim, Juhwan Yoo, Kyungdo Han, Maurizio Fava, David Mischoulon, Mi Jin Park, Hong Jin Jeon

**Affiliations:** ^1^Department of Psychiatry, Hanyang University Hospital, Seoul, South Korea; ^2^Department of Biomedicine and Health Science, The Catholic University of Korea, Seoul, South Korea; ^3^Department of Statistics and Actuarial Science, Soongsil University, Seoul, South Korea; ^4^Depression Clinical and Research Program, Massachusetts General Hospital and Harvard Medical School, Boston, MA, United States; ^5^Department of Psychiatry, Depression Center, Samsung Medical Center, Sungkyunkwan University School of Medicine, Seoul, South Korea; ^6^Department of Health Sciences and Technology, Department of Medical Device Management and Research, and Department of Clinical Research Design and Evaluation, Samsung Advanced Institute for Health Sciences and Technology (SAIHST), Sungkyunkwan University, Seoul, South Korea

**Keywords:** alcohol consumption, depression, lifestyle factors, physical activity, smoking—adverse effects, middle aged women

## Abstract

**Background:** Changes in lifestyle factors are known to affect mood. However, there is insufficient evidence supporting the association between smoking, alcohol consumption, physical activity and depression in middle-aged women who are likely to experience rapid hormonal changes.

**Methods:** We used a nationwide database of medical records in South Korea. 901,721 premenopausal and 943,710 postmenopausal women aged 40 years or older included in this study. Information on smoking, alcohol consumption, physical activity was identified from health examination data and followed up for the occurrence of depression using claims data.

**Results:** Compared with never-smokers, ex-smokers and current smokers among premenopausal and postmenopausal women showed an increased risk of depression in a dose-dependent manner (aHR 1.13 for ex-smokers; aHR 1.23 for current smokers). Compared with non-drinkers, mild drinkers showed a decreased risk of depression (aHR 0.98 for premenopausal women; aHR 0.95 for postmenopausal women), and heavy drinkers showed an increased risk of depression both among premenopausal (aHR 1.20) and postmenopausal women (aHR 1.05). The risk of depression due to smoking and heavy alcohol consumption was higher in premenopausal women than in postmenopausal women. Compared with those who had not engaged in regular physical activity, those who had engaged showed a decreased risk of depression both among premenopausal (aHR 0.96) and postmenopausal women (aHR 0.95).

**Conclusions:** Smoking and heavy alcohol consumption increased the risk of depression, and the increased risk was prominent in premenopausal than in postmenopausal women. Regular physical activity decreased the risk of depression both in premenopausal and postmenopausal women.

## Introduction

Depression is a common mental illness associated with significant morbidity (1). Depression is characterized by symptoms of depressed mood, decreased interest, motivation and cognition, accompanied by vegetative symptoms including changes in appetite or sleep. Depressive symptoms can significantly impair individual functioning and sometimes lead to suicide attempts, with depression associated with 60–70% of all suicides (2). A previous study using a large representative sample of the South Korean population reported that the overall prevalence of depression was approximately 5.3% with a higher prevalence among females (6.81%) than in males (3.88%) (3).

Globally, the prevalence of depression is higher in women than in men (4–6). The sex-based differences in depression (7) may be attributed to estrogen, a major female sex hormone, although affected by various socioeconomic factors. Unlike men, women experience certain forms of depression such as premenstrual dysphoric disorder, and postpartum depression that occur during periods of rapid changes in estrogen levels (8). In addition, studies have reported that removal of one or both ovaries via oophorectomy increases the risk of depression (9, 10). In animal models, oophorectomy increased depressive behaviors, which were reversed by the administration of estrogen (11, 12). Women's reproductive aging cycle includes the reproductive stage, menopausal transition stage and postmenopausal stage according to changes in the levels of female sex hormones including estrogen (13). Although the period of menopausal transition varies individually, on average, the level of estrogen rapidly decreases from 2 years before to 2 years after the final menstrual period, and thereafter remains very low (14). Therefore, the risk of depression associated with decreased levels of estrogen may occur in middle-aged women in their 40s and later ages considering the average age of menopause in women (15).

Smoking, alcohol consumption, and physical inactivity are established unhealthy lifestyle factors, which increase the risk of cardiovascular and metabolic diseases (16–19). Evidence demonstrates the association between these lifestyle factors and depression. Smoking is very common in patients with mental disorders (20). Previous studies have reported mixed effects of smoking on mood. A previous study reported antidepressant and anxiolytic effects of smoking (21), and another study suggested that nicotine use by smokers improves adaptation to stressful situations (22). However, despite a temporary increase in mood immediately after smoking, throughout the day, the mood was worse than that of non-smokers, and the diurnal variation was larger in smokers (23). Further, a prospective cohort study showed that smokers had an increased risk of major depressive disorder by about 93% compared with non-smokers (24). A number of studies have shown a positive association between alcohol consumption and depression (25–29). Studies reported that the amount of alcohol consumption affects depression more than the act of drinking, and a strong association with depression was found in heavy drinkers (27, 28). In addition, the association between alcohol consumption and depression was stronger in women than in men (26, 27, 29). Studies have consistently reported a protective effect of physical activity on depression. A case-control study involving older men and women showed that physical activity had a protective effect on depressive disorders with an odds ratio (OR) of 0.55 (30). A cohort study of middle-aged and elderly individuals showed that greater physical activity prevented depression (OR = 0.83) (31). Further, a large-sample British prospective cohort study showed a bidirectional association between physical activity and depression, suggesting that physical activity has a protective effect on depression (OR = 0.72) and individuals with depression are unlikely to meet the recommended levels of physical activity (OR = 1.79) (32).

In this study, we investigated the association between smoking, alcohol consumption, physical activity and depression in middle-aged women, with a particular focus on the differences between premenopausal and postmenopausal women. We hypothesized that (1) smoking and alcohol consumption increase the risk of depression; (2) regular physical activity decreases the risk of depression; (3) a discrepancy in these associations exists between premenopausal and postmenopausal women.

## Methods

### Data Source

This study used the National Health Insurance Sharing Service (NHISS) database of National Health Insurance Service (NHIS) of South Korea (33, 34). NHIS is a public institution responsible for operating a mandatory universal health insurance program. Nearly 97% of the total South Korean population is enrolled in this service, while the remaining 3% is covered by the Medical Aid Program. The NHISS database contains not only the medical services claims data such as admission, emergency room visits, ambulatory care visits, and pharmaceutical services but also health checkup data. NHISS data are anonymized to protect the privacy of individuals.

We also used the National Cancer Screening Program (NCSP) database (35). The NCSP includes screening of all individuals for stomach, liver, colorectal, breast, and cervical cancers based on age. In addition, subjects respond to self-questionnaires on lifestyle factors such as smoking, alcohol consumption, and physical activity. All South Korean women aged 40 years or older are recommended screening for breast and cervical cancers. Although the screening program is voluntary, the participation rate is as high as approximately 70% (36).

The NHISS and NCSP data are linked and anonymized to protect individual privacy. The study protocol was approved by the Institutional Review Board of the Samsung Medical Center (No. SMC 2020-10-005).

### Case Identification

**Figure 1:**
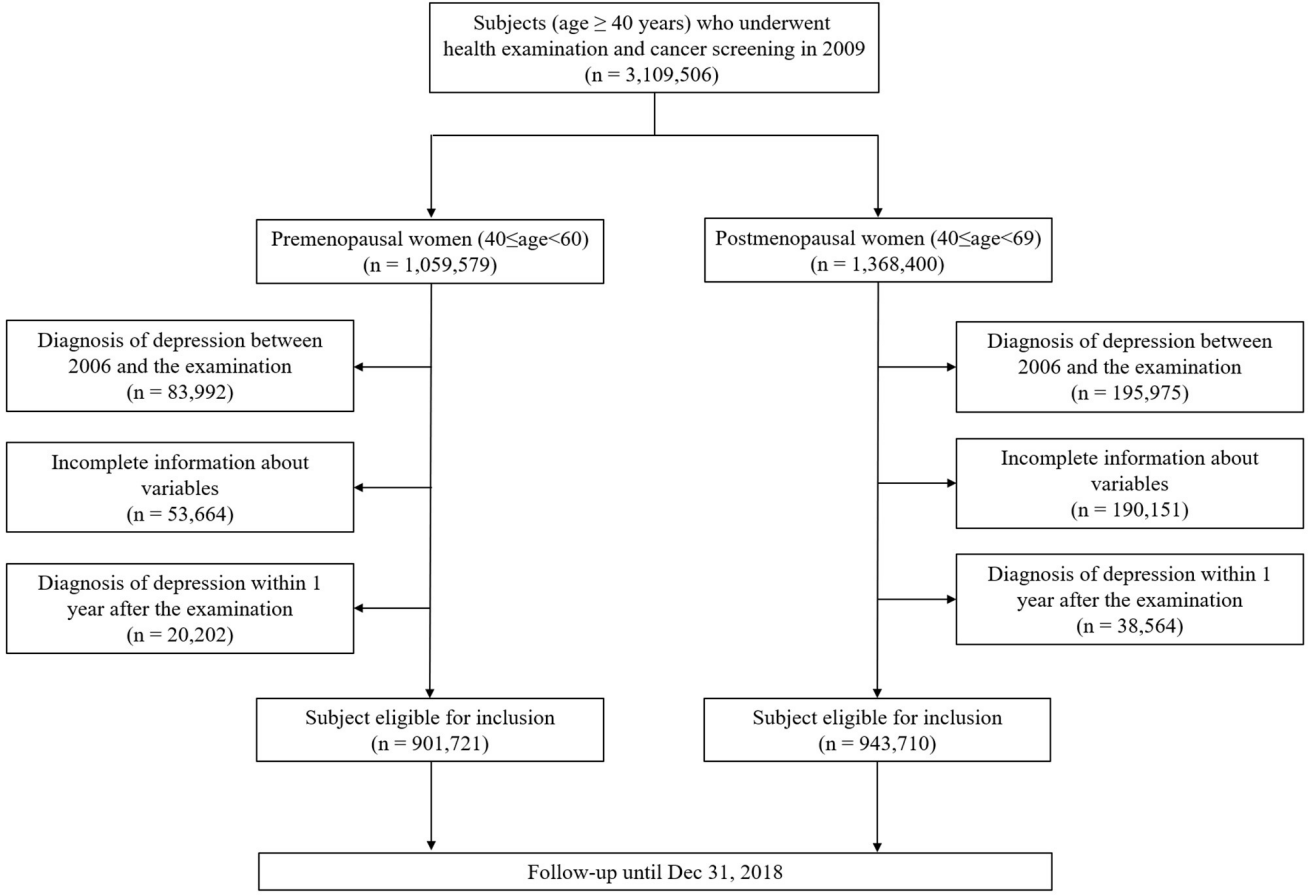
Flow chart of subject identification.

For the case identification, first, we used NCSP data. We identified subjects' lifestyle factors including smoking, alcohol consumption and physical activity from self-questionnaire in the health examination data, and menopausal status and female reproductive factors from data of screening for breast and cervical cancer data. A total of 3,109,506 female subjects aged 40 years or older underwent health examination and screening for breast and cervical cancer on the same day from January 1, 2009 to December 31, 2009. Among them, 1,059,579 were premenopausal women aged 40–59 years and 1,368,400 were postmenopausal women aged 40–69 years. Then, using NHISS data, we excluded people with a history of depressive disorder before the examination (83,992 and 195,975 cases of premenopausal and postmenopausal women, respectively) to establish the first diagnosis of depressive disorder. Those with incomplete information about variables were also excluded (53,664 and 190,151 cases of premenopausal and postmenopausal women, respectively). We also excluded 20,202 premenopausal and 38,564 postmenopausal women who were diagnosed with depressive disorder within 1 year after the examination to eliminate the effect of a temporary increase in the diagnosis of depression following the examination. Finally, 901,721 premenopausal and 943,710 postmenopausal women were eligible and included in the study. We reviewed their medical records using NHISS data until December 31, 2018 (Figure 1).

### Smoking Status

We extracted data involving smoking status, duration of smoking, and the level of smoking among subjects from the self-administrated questionnaire conducted on the day of health examination. Based on smoking status, subjects were categorized into never smokers, ex-smokers, and current smokers. The level of smoking was measured in terms of cigarettes smoked per day and pack-years.

### Alcohol Consumption

The number of drinking days per week and the amount of daily drinking were determined from the self-administrated questionnaire administered on the day of health examination. We categorized alcohol users according to the amount of alcohol consumption: mild (up to 1.5 drinks a day), moderate (1.5–3 drinks a day) and heavy (more than 3 drinks a day).

### Physical Activity

In the self-questionnaire during health examination, subjects' physical activity was measured based on their response indicating the number of days a week of intense physical exercise lasting more than 20 mins, moderate intensity exercise of more than 30 mins and mild intensity workout of more than 30 mins. Regular exercise was operationally defined as moderate physical activity of more than 30 mins at least 5 times a week or intense physical activity of more than 20 mins at least 3 times a week.

### Outcomes

The main outcome was the diagnosis of depressive disorder during the follow-up period identified from NHISS claims data. Depressive disorder (F32 and F33) was defined according to the International Statistical Classification of Disease and Related Health Problems 10th revision (ICD-10) and the diagnostic codes were entered by clinicians when they see a patient.

### Subgroup

We performed subgroup analysis to identify the association between lifestyle factors and depression according to the diagnostic history of bipolar disorder. The subgroup with bipolar disorder was defined as those who had diagnosed with bipolar disorder (F30 and F31) as a primary diagnosis during entire research period (from January 1, 2009 to December 31, 2018).

### Covariates

We calculated body mass index (BMI) using weight and height information. Comorbid physical illnesses including hypertension, diabetes mellitus, and dyslipidemia were identified from past medical history based on ICD-10 codes. We identified the age at menarche, age at menopause, parity, duration of oral contraceptive (OC) use, duration of breast feeding, and duration of hormone replacement therapy (HRT) as female reproductive factors using the self-administered questionnaire data. The duration of fertility was calculated as the interval between age at menarche and age at menopause.

### Statistical Analyses

We presented the baseline characteristics (i.e., personal information, physical status, smoking status, alcohol consumption, regular physical activity, female reproductive factors, comorbid physical illnesses and comorbid psychiatric illnesses) with continuous variables as mean ± standard deviation (SD) and categorical variables as numbers and percentages. Student's t-tests and chi-square tests were used to compare the differences in baseline factors between depression group and non-depression group. Cox proportional hazards regression analyses were conducted to identify the association between smoking, alcohol consumption, physical activity and diagnosis of depression. Hazard ratios (HR) were presented to show the magnitude of risk of depression according to the variables. Statistical analyses were performed using SAS version 9.4 (SAS institute Inc., Cary, NC, USA).

## Results

### Baseline Characteristics of the Study Subjects

Table 1 shows the baseline characteristics of the study subjects. The mean age was older in those diagnosed with depression both among premenopausal (depression group: 45.77, SD 3.99 vs. non-depression group: 44.81, SD 3.91) and postmenopausal women (depression group: 59.31, SD 5.81 vs. non-depression group: 57.99, SD 5.78). There were more never-smokers in the non-depression groups, and more ex-smokers and current smokers in the depression group both in premenopausal and postmenopausal women. Regarding alcohol consumption, there were more mild alcohol users in the non-depression group and more heavy alcohol users in the depression group both in premenopausal and postmenopausal women. There were more non-alcohol users in the non-depression group in premenopausal women, and more in the depression group in postmenopausal women. The proportion of those who had engaged in regular physical activity was higher in the non-depression group than in the depression group among postmenopausal women, while there was no significant difference between groups among premenopausal women.

**Table 1:** Baseline characteristics of study subjects.

	**Premenopausal women**			**Postmenopausal women**		
	**Depression**		***p***	**Depression**		***p***
	**No (*n* = 737,575)**	**Yes (*n* = 164,146)**		**No (*n* = 674,171)**	**Yes (*n* = 269,539)**	
Age (years)	44.81 ± 3.91	45.77 ± 3.99	<0.0001	57.99 ± 5.78	59.31 ± 5.81	<0.0001
BMI (kg/m^2^)	23.2 ± 3.05	23.36 ± 3.11	<0.0001	24.18 ± 3.09	24.34 ± 3.11	<0.0001
Systolic BP (mmHg)	117.27 ± 14.35	117.46 ± 14.35	<0.0001	124.42 ± 15.93	124.69 ± 15.75	<0.0001
Diastolic BP (mmHg)	73.14 ± 9.97	73.4 ± 9.95	<0.0001	76.59 ± 10.19	76.69 ± 10.05	<0.0001
Fasting glucose (mg/dL)	93.54 ± 17.42	94.15 ± 19.5	<0.0001	98.77 ± 23.12	99.54 ± 24.67	<0.0001
Total cholesterol (mg/dL)	191.8 ± 38.36	193.34 ± 40.82	<0.0001	209.41 ± 43.03	208.27 ± 44.37	<0.0001
HDL (mg/dL)	60.45 ± 35.54	60.51 ± 36.34	0.531	58.23 ± 34.41	57.85 ± 34.65	<0.0001
LDL (mg/dL)	114.5 ± 71.61	115.02 ± 60.57	0.006	128.54 ± 70.74	126.99 ± 72.36	<0.0001
Smoking status			<0.0001			<0.0001
Never	706,116 (95.73)	154,513 (94.13)		652,186 (96.74)	259,511 (96.28)	
Ex-smoker	10,001 (1.36)	2,691 (1.64)		6,016 (0.89)	2,526 (0.94)	
Current smoker	21,458 (2.91)	6,942 (4.23)		15,969 (2.37)	7,502 (2.78)	
Alcohol consumption^a^			<0.0001			<0.0001
None	528,694 (71.68)	117,543 (71.61)		57,6493 (85.51)	234,015 (86.82)	
Mild	180,131 (24.42)	38,390 (23.39)		83,913 (12.45)	30,044 (11.15)	
Moderate	21,155 (2.87)	5,801 (3.53)		9,866 (1.46)	3,862 (1.43)	
Heavy	7,595 (1.03)	2,412 (1.47)		3,899 (0.58)	1,618 (0.60)	
Regular physical activity	127,624 (17.30)	28,096 (17.12)	0.070	135,839 (20.15)	51,960 (19.28)	<0.0001
Hypertension	99,390 (13.48)	26,068 (15.88)	<0.0001	262,002 (38.86)	116,889 (43.37)	<0.0001
Diabetes mellitus	20,963 (2.84)	5,798 (3.53)	<0.0001	49,534 (7.35)	22,687 (8.42)	<0.0001
Dyslipidemia	78,424 (10.63)	21,189 (12.91)	<0.0001	216,573 (32.12)	94,337 (35.00)	<0.0001
Stroke	1,457 (0.40)	457 (0.55)	<0.0001	5,204 (1.20)	3,048 (1.68)	<0.0001
Cardiovascular disease	2,945 (0.80)	1,008 (1.21)	<0.0001	14,748 (3.39)	9,156 (5.04)	<0.0001
Parity			<0.0001			<0.0001
Nulliparity	31,140 (4.22)	6,328 (3.86)		18,817 (2.79)	6,776 (2.51)	
1	97,371 (13.2)	22,391 (13.64)		49,279 (7.31)	16,511 (6.13)	
≥ 2	609,064 (82.58)	135,427 (82.50)		606,075 (89.90)	246,252 (91.36)	
Duration of breast feeding (months)			<0.0001			<0.0001
Never	183,268 (24.85)	36,240 (22.08)		53,995 (8.01)	16,866 (6.26)	
< 6	197,576 (26.79)	42,563 (25.93)		132,905 (19.71)	46,630 (17.3)	
6–12	221,624 (30.05)	57,254 (34.88)		434,071 (64.39)	188,405 (69.90)	
≥ 12	135,107 (18.32)	28,089 (17.11)		53,200 (7.89)	176,38 (6.54)	
Duration of OC use (years)			<0.0001			<0.0001
Never	617,748 (83.75)	134,076 (81.68)		543,673 (80.64)	215,315 (79.88)	
< 1	66,813 (9.06)	16,254 (9.90)		66,260 (9.83)	26,567 (9.86)	
≥ 1	23,155 (3.14)	6,342 (3.86)		41,036 (6.09)	18,166 (6.74)	
Unknown	29,859 (4.05)	7,474 (4.55)		23,202 (3.44)	9,491 (3.52)	
Age at menarche (years)	15.05 ± 1.64	15.31 ± 1.71	<0.0001	16.26 ± 1.83	16.48 ± 1.84	<0.0001
Age at menopause (years)	–	–	–	50.26 ± 3.79	50.16 ± 3.97	<0.0001
Duration of fertility (years)	–	–	–	34 ± 4.13	33.68 ± 4.35	<0.0001
Duration of HRT (years)			–			<0.0001
Never	–	–		560,433 (83.13)	216,136 (80.19)	
< 2	–	–		67,186 (9.97)	30,489 (11.31)	
2–5	–	–		27,527 (4.08)	12,565 (4.66)	
≥ 5	–	–		19,025 (2.82)	10,349 (3.84)	
Diagnosis of bipolar disorder	989 (0.13)	874 (0.53)	<0.0001	896 (0.13)	929 (0.34)	<0.0001
Substance-related disorders	3,814 (0.52)	2,231 (1.36)	<0.0001	3,258 (0.48)	2,263 (0.84)	<0.0001

*Data are expressed as the mean ± standard deviation, SD, or n (%)*.

*BMI, body mass index; BP, blood pressure; HDL, high-density lipoprotein; LDL, low-density lipoprotein; OC, oral contraceptives; HRT, hormone replacement therapy*.

a *Alcohol consumption: mild = up to 15 g (equivalent to 1.5 drinks) a day; moderate = 15 g to 30 g a day; heavy = more than 30g (equivalent to 3 drinks) a day*.

### Incidence of Depressive Disorder

Among premenopausal women, the average length of follow-up was 7.54 years (SD, 1.98), 164,146 patients were newly diagnosed with a depressive disorder during the follow-up period, and the incidence rate was 24.1 per 1,000 person-years.

Among postmenopausal women, the average length of follow-up was 7.07 years (SD, 2.45), 269,539 patients were newly diagnosed with a depressive disorder during the follow-up period, and the incidence rate was 40.4 per 1,000 person-years.

### Hazard Ratios of Smoking, Alcohol Consumption, and Regular Physical Activity for Occurrence of Depression

Table 2 presents HRs of depression according to the status of smoking, alcohol consumption, and physical activity. Compared with never-smokers, ex-smokers and current smokers showed an increased HR both among premenopausal (aHR 1.28, 95% CI 1.23–1.33 for ex-smokers and aHR 1.40, 95% CI 1.36–1.43 for current smokers) and postmenopausal women (aHR 1.13, 95% CI 1.09–1.17 for ex-smokers and aHR 1.23, 95% CI 1.20–1.26 for current smokers). Compared with non-alcohol users, mild alcohol users showed a decreased risk of depression (aHR 0.98, 95% CI 0.97–0.99), whereas moderate (aHR 1.12, 95% CI 1.09–1.15) and heavy alcohol users (aHR 1.20, 95% CI 1.15–1.25) showed an increased risk of depression among premenopausal women. Among postmenopausal women, heavy alcohol users showed an increased risk of depression compared with non-alcohol users (aHR 1.05, 95% CI 1.00–1.10). A decreased risk of depression was found among those engaged in regular physical activity: premenopausal (aHR 0.96, 95% CI 0.95–0.97) and postmenopausal women (aHR 0.95, 95% CI 0.94–0.96).

**Table 2:** Hazard ratios of lifestyle factors associated with the occurrence of depression.

	**Hazard ratio (95% confidence interval)**				
	**Premenopausal women**		**Postmenopausal women**		
	**Crude**	**Model**	**Crude**	**Model 1**	**Model 2**
Smoking status					
Never	1 (Ref.)	1 (Ref.)	1 (Ref.)	1 (Ref.)	1 (Ref.)
Ex-smoker	1.21 (1.16, 1.26)	1.28 (1.23, 1.33)	1.06 (1.02, 1.10)	1.13 (1.09, 1.17)	1.13 (1.08, 1.17)
Current smoker	1.43 (1.39, 1.46)	1.40 (1.36, 1.43)	1.16 (1.14, 1.19)	1.23 (1.20,1.26)	1.23 (1.20, 1.26)
Alcohol consumption^a^					
None	1 (Ref.)	1 (Ref.)	1 (Ref.)	1 (Ref.)	1 (Ref.)
Mild	0.96 (0.95, 0.97)	0.98 (0.97, 0.99)	0.90 (0.89, 0.91)	0.95 (0.94, 0.96)	0.95 (0.94, 0.96)
Moderate	1.21 (1.18, 1.24)	1.12 (1.09, 1.15)	0.97 (0.94, 1.00)	1.01 (0.98, 1.05)	1.02 (0.98,1.05)
Heavy	1.38 (1.32, 1.43)	1.20 (1.15, 1.25)	1.02 (0.97, 1.07)	1.05 (1.00, 1.10)	1.05 (1.00, 1.10)
Regular physical activity					
No	1 (Ref.)	1 (Ref.)	1 (Ref.)	1 (Ref.)	1 (Ref.)
Yes	0.99 (0.97, 1.00)	0.96 (0.95, 0.97)	0.95 (0.95, 0.96)	0.95 (0.94, 0.96)	0.95 (0.94, 0.96)

*Model: adjusted for age at menarche, parity, duration of breast feeding, history of oral contraceptive use, age, smoking status, alcohol consumption, regular exercise, body mass index, diabetes mellitus, hypertension, and dyslipidemia*.

*Model 1: adjusted for age at menarche, age at menopause, parity, duration of breast feeding, history of oral contraceptive use, duration of hormone replacement therapy, age, smoking status, alcohol consumption, regular exercise, body mass index, diabetes mellitus, hypertension, and dyslipidemia*.

*Model 2: adjusted for duration of fertility instead of age at menarche and menopause in Model 1*.

a *Alcohol consumption: mild = up to 15 g (equivalent to 1.5 drinks) a day; moderate = 15 g to 30 g a day; heavy = more than 30 g (equivalent to 3 drinks) a day*.

Supplementary Table 1 shows the associations between lifestyle factors and risk of depression in the subgroup without history of bipolar disorder, and the results were similar with the main outcome. While, Supplementary Table 2 shows the associations between lifestyle factors and risk of depression in the subgroup with history of bipolar disorder. Compared with never-smokers, current smokers showed an increased HR both among premenopausal (aHR 1.44, 95% CI 1.12–1.86) and postmenopausal women (aHR 1.46, 95% CI 1.06–2.00).

Table 3 shows the HRs stratified according to ever smoking, heavy alcohol use, and regular physical activity. Compared with never-smoking, non-heavy alcohol use, and regular physical activity, the lowest risk of depression was found both in premenopausal (aHR 0.74, 95% CI 0.69–0.79) and postmenopausal women (aHR 0.85, 95% CI 0.80–0.90) who ever smoked, and engaged in non-heavy alcohol use and regular physical activity, while those who never smoked, indulged in heavy alcohol use and not engaged in regular physical activity showed the highest risk of depression among premenopausal (aHR 1.23, 95% CI 1.11–1.36) and postmenopausal women (aHR 1.16, 95% CI 1.03–1.30).

**Table 3:** Hazard ratios of combinations of lifestyle factors associated with the occurrence of depression.

			**Premenopausal women**		**Postmenopausal women**		
			**Crude**	**Model**	**Crude**	**Model 1**	**Model 2**
**Ever smoking**	**Heavy drinking**	**Regular physical activity**	**Hazard ratio (95% confidence interval)**				
No	No	Yes	1 (Ref.)	1 (Ref.)	1 (Ref.)	1 (Ref.)	1 (Ref.)
No	No	No	1.06 (0.98, 1.13)	1.07 (1.00, 1.15)	1.10 (1.03, 1.17)	1.09 (1.03, 1.16)	1.09 (1.03, 1.16)
No	Yes	Yes	1.16 (0.97, 1.40)	1.16 (0.96, 1.39)	0.96 (0.76, 1.21)	1.06 (0.84, 1.33)	1.05 (0.83, 1.33)
No	Yes	No	1.22 (1.11, 1.35)	1.23 (1.11, 1.36)	1.06 (0.94, 1.19)	1.16 (1.03, 1.30)	1.15 (1.02, 1.30)
Yes	No	Yes	0.74 (0.69, 0.79)	0.74 (0.69, 0.79)	0.89 (0.84, 0.95)	0.85 (0.80, 0.90)	0.85 (0.80, 0.90)
Yes	No	No	0.75 (0.70, 0.80)	0.76 (0.72, 0.82)	0.93 (0.88, 0.99)	0.90 (0.84, 0.95)	0.89 (0.84, 0.95)
Yes	Yes	Yes	0.92 (0.81, 1.04)	0.88 (0.78, 0.99)	0.89 (0.78, 1.01)	0.91 (0.80, 1.04)	0.91 (0.80, 1.05)
Yes	Yes	No	0.99 (0.91, 1.08)	0.96 (0.89, 1.05)	0.95 (0.87, 1.03)	0.95 (0.87, 1.03)	0.95 (0.87,1.04)

*Model: adjusted for age at menarche, parity, duration of breast feeding, history of oral contraceptive use, age, smoking status, alcohol consumption, regular exercise, body mass index, diabetes mellitus, hypertension, and dyslipidemia*.

*Model 1: adjusted for age at menarche, age at menopause, parity, duration of breast feeding, history of oral contraceptive use, duration of hormone replacement therapy, age, smoking status, alcohol consumption, regular exercise, body mass index, diabetes mellitus, hypertension, and dyslipidemia*.

*Model 2: adjusted for duration of fertility instead of age at menarche and menopause in Model 1*.

### Dose-Response Relationships Between Smoking, Alcohol Consumption, and Regular Physical Activity With Depression

Table 4 shows the dose-response relationships between lifestyle factors and the risk of depression. We found a strong positive dose-response relationship between smoking and depression. Compared with never smokers, current smokers who smoked more than 20 cigarettes a day showed the highest HRs (aHR 1.65, 95% CI 1.57–1.75 for premenopausal women; aHR 1.34, 95% CI 1.28–1.41 for postmenopausal women). Among premenopausal women, compared with never smokers, current smokers who had been smoking for over 20 years showed the highest risk of depression (aHR 1.45, 95% CI 1.38–1.53). While among postmenopausal women, current smokers who smoked for 10–20 years showed the highest risk of depression (aHR 1.27, 95% CI 1.22–1.32). Compared with never smokers, current smokers who had smoked 20 pack-years or more showed the highest risk of depression both among premenopausal (aHR 1.66, 95% CI 1.53–1.80) and postmenopausal women (aHR 1.32, 95% CI 1.25–1.38).

**Table 4:** The dose-response relationships between lifestyle factors and the risk of depression.

	**Hazard ratio (95% confidence interval)**				
	**Premenopausal women**		**Postmenopausal women**		
	**Crude**	**Model**	**Crude**	**Model 1**	**Model 2**
**Smoking**					
Level of smoking (cigarettes/day)					
Never smoker	1 (Ref.)	1 (Ref.)	1 (Ref.)	1 (Ref.)	1 (Ref.)
Ex-smoker / < 10	1.14 (1.08, 1.20)	1.23 (1.17, 1.29)	1.02 (0.96, 1.08)	1.11 (1.04, 1.18)	1.10 (1.04, 1.17)
Ex-smoker / < 20	1.26 (1.18,1.34)	1.31 (1.23, 1.40)	1.02 (0.96, 1.09)	1.10 (1.03, 1.17)	1.09 (1.03, 1.17)
Ex-smoker / ≥ 20	1.45 (1.30, 1.61)	1.42 (1.27, 1.58)	1.23 (1.13, 1.35)	1.25 (1.14, 1.36)	1.24 (1.14,1.36)
Current smoker / < 10	1.24 (1.20, 1.29)	1.26 (1.21, 1.31)	1.08 (1.04, 1.13)	1.14 (1.10, 1.19)	1.14 (1.10, 1.19)
Current smoker / < 20	1.46 (1.42, 1.51)	1.43 (1.38, 1.48)	1.16 (1.12, 1.20)	1.24 (1.20, 1.28)	1.24 (1.20, 1.28)
Current smoker / ≥ 20	1.81 (1.72, 1.91)	1.65 (1.57, 1.75)	1.30 (1.24, 1.36)	1.34 (1.28, 1.41)	1.34 (1.28, 1.41)
Duration of smoking (years)					
Never smoker	1 (Ref.)	1 (Ref.)	1 (Ref.)	1 (Ref.)	1 (Ref.)
Ex-smoker / < 10	1.17 (1.11, 1.23)	1.26 (1.20, 1.33)	1.01 (0.95,1.08)	1.12 (1.06, 1.20)	1.12 (1.05, 1.19)
Ex-smoker / < 20	1.26 (1.17, 1.34)	1.31 (1.22, 1.40)	1.03 (0.96, 1.10)	1.11 (1.04, 1.19)	1.11 (1.04, 1.19)
Ex-smoker / ≥ 20	1.36 (1.19, 1.55)	1.25 (1.10, 1.44)	1.17 (1.08, 1.26)	1.16 (1.07, 1.25)	1.15 (1.07, 1.24)
Current smoker / < 10	1.38 (1.32, 1.44)	1.38 (1.33, 1.44)	1.05 (0.99, 1.11)	1.18 (1.12, 1.25)	1.18 (1.12, 1.25)
Current smoker / < 20	1.39 (1.34, 1.44)	1.38 (1.33, 1.43)	1.15 (1.10, 1.20)	1.27 (1.22, 1.32)	1.27 (1.22, 1.32)
Current smoker / ≥ 20	1.56 (1.49, 1.64)	1.45 (1.38, 1.53)	1.22 (1.18, 1.26)	1.22 (1.18, 1.26)	1.22 (1.18, 1.26)
Pack-years of smoking					
Never smoker	1 (Ref.)	1 (Ref.)	1 (Ref.)	1 (Ref.)	1 (Ref.)
Ex-smoker / < 20	1.20 (1.15, 1.25)	1.27 (1.23, 1.33)	1.03 (0.99, 1.07)	1.11 (1.07, 1.16)	1.11 (1.06, 1.16)
Ex-smoker / ≥ 20	1.54 (1.24, 1.90)	1.38 (1.12, 1.71)	1.31 (1.17, 1.46)	1.26 (1.13, 1.41)	1.26 (1.12, 1.41)
Current smoker / < 20	1.39 (1.36, 1.43)	1.38 (1.34, 1.41)	1.13 (1.10, 1.16)	1.21 (1.18, 1.24)	1.21 (1.18, 1.24)
Current smoker / ≥ 20	1.88 (1.74, 2.04)	1.66 (1.53, 1.80)	1.34 (1.27, 1.41)	1.32 (1.25, 1.38)	1.31 (1.25, 1.38)
**Alcohol consumption**					
Drinking days per week					
Non-drinker	1 (Ref.)	1 (Ref.)	1 (Ref.)	1 (Ref.)	1 (Ref.)
< 3	0.97 (0.96, 0.98)	0.98 (0.97, 0.99)	0.89 (0.88, 0.91)	0.95 (0.94, 0.96)	0.95 (0.94, 0.96)
< 5	1.16 (1.13, 1.19)	1.08 (1.06, 1.11)	0.95 (0.92, 0.98)	0.99 (0.96, 1.02)	0.99 (0.97, 1.02)
≥ 5	1.32 (1.27, 1.37)	1.16 (1.12, 1.21)	1.04 (0.99, 1.08)	1.05 (1.01, 1.09)	1.05 (1.01, 1.09)
Amount of daily alcohol consumption (drinks)					
Non-drinker	1 (Ref.)	1 (Ref.)	1 (Ref.)	1 (Ref.)	1 (Ref.)
≤ 4	0.95 (0.94, 0.96)	0.97 (0.96, 0.98)	0.90 (0.89, 0.91)	0.95 (0.93, 0.96)	0.95 (0.94, 0.96)
5–7	1.11 (1.09, 1.13)	1.07 (1.05, 1.09)	0.93 (0.91, 0.96)	1.00 (0.97, 1.02)	1.00 (0.98, 1.02)
8–14	1.24 (1.20, 1.29)	1.15 (1.11, 1.19)	1.01 (0.96, 1.06)	1.06 (1.01, 1.11)	1.06 (1.01, 1.12)
>14	1.36 (1.22, 1.50)	1.19 (1.07, 1.32)	0.97 (0.85, 1.11)	0.99 (0.87, 1.13)	1.00 (0.87, 1.13)
**Physical activity**					
Days of walking per week					
0	1.11 (1.09, 1.13)	1.05 (1.03, 1.07)	1.07 (1.05, 1.08)	1.03 (1.02, 1.05)	1.03 (1.02, 1.05)
1	1 (Ref.)	1 (Ref.)	1 (Ref.)	1 (Ref.)	1 (Ref.)
2	0.96 (0.94, 0.98)	0.97 (0.95, 0.99)	0.98 (0.96, 1.00)	0.98 (0.96, 1.00)	0.98 (0.96, 1.00)
3	0.98 (0.96, 1.00)	0.97 (0.95, 0.99)	0.96 (0.94, 0.98)	0.95 (0.94, 0.97)	0.95 (0.94, 0.97)
4	0.98 (0.95, 1.00)	0.95 (0.93, 0.98)	0.97 (0.95, 0.99)	0.95 (0.93, 0.97)	0.95 (0.94, 0.97)
5	0.90 (0.88, 0.92)	0.89 (0.87, 0.91)	0.92 (0.91, 0.94)	0.91 (0.90, 0.93)	0.91 (0.90, 0.93)
6	0.96 (0.94, 0.99)	0.93 (0.91, 0.95)	0.94 (0.92, 0.96)	0.92 (0.90, 0.94)	0.92 (0.90, 0.94)
7	1.02 (1.00, 1.05)	0.97 (0.95, 0.99)	1.01 (1.00, 1.03)	0.95 (0.94, 0.97)	0.96 (0.94, 0.97)
Days of moderate intensity physical activities per week					
0	1.10 (1.09, 1.12)	1.06 (1.04, 1.08)	1.07 (1.06, 1.09)	1.03 (1.01, 1.04)	1.03 (1.01, 1.04)
1	1 (Ref.)	1 (Ref.)	1 (Ref.)	1 (Ref.)	1 (Ref.)
2	1.00 (0.98, 1.03)	1.00 (0.97, 1.02)	0.96 (0.94, 0.98)	0.96 (0.94, 0.98)	0.96 (0.94, 0.98)
3	1.02 (1.00, 1.05)	1.00 (0.98, 1.02)	0.99 (0.97, 1.01)	0.97 (0.95, 0.99)	0.97 (0.95, 0.99)
4	1.04 (1.01, 1.07)	1.01 (0.98, 1.04)	1.00 (0.97, 1.02)	0.98 (0.96, 1.01)	0.98 (0.96, 1.01)
5	1.01 (0.98, 1.04)	0.97 (0.94, 1.00)	0.98 (0.96, 1.00)	0.95 (0.93, 0.98)	0.95 (0.93, 0.98)
6	1.08 (1.03, 1.12)	1.00 (0.96, 1.05)	1.03 (1.00, 1.06)	0.98 (0.95, 1.01)	0.98 (0.95, 1.01)
7	1.19 (1.15, 1.23)	1.08 (1.04, 1.12)	1.06 (1.04, 1.09)	0.98 (0.96, 1.00)	0.98 (0.96, 1.01)
Days of vigorous intensity physical activities per week					
0	1.07 (1.05, 1.09)	1.06 (1.04, 1.07)	1.09 (1.07, 1.10)	1.03 (1.02, 1.05)	1.03 (1.02, 1.05)
1	1 (Ref.)	1 (Ref.)	1 (Ref.)	1 (Ref.)	1 (Ref.)
2	1.02 (0.99, 1.04)	1.01 (0.98, 1.03)	0.97 (0.95, 0.99)	0.97 (0.95, 0.99)	0.97 (0.95, 0.99)
3	1.02 (0.99, 1.04)	1.00 (0.98, 1.03)	0.99 (0.97, 1.01)	0.97 (0.95, 0.99)	0.97 (0.95, 0.99)
4	1.03 (0.99, 1.06)	1.00 (0.97, 1.04)	0.99 (0.97, 1.02)	0.98 (0.95, 1.00)	0.98 (0.95, 1.00)
5	0.99 (0.96, 1.02)	0.97 (0.93, 1.00)	0.98 (0.95, 1.01)	0.95 (0.93, 0.98)	0.95 (0.93, 0.98)
6	1.07 (1.02, 1.13)	1.00 (0.96, 1.05)	1.06 (1.02, 1.09)	1.00 (0.97, 1.03)	1.00 (0.97, 1.04)
7	1.22 (1.17, 1.27)	1.10 (1.05, 1.14)	1.10 (1.07, 1.13)	1.01 (0.98, 1.04)	1.01 (0.99, 1.04)

*Model: adjusted for age at menarche, parity, duration of breast feeding, history of oral contraceptive use, age, smoking status, alcohol consumption, regular exercise, body mass index, diabetes mellitus, hypertension, and dyslipidemia*.

*Model 1: adjusted for age at menarche, age at menopause, parity, duration of breast feeding, history of oral contraceptive use, duration of hormone replacement therapy, age, smoking status, alcohol consumption, regular exercise, body mass index, diabetes mellitus, hypertension, and dyslipidemia*.

*Model 2: adjusted for duration of fertility instead of age at menarche and menopause in Model 1*.

Regarding alcohol consumption, compared with non-drinkers, those who consumed alcohol during fewer than 3 days a week showed a decreased risk of depression (aHR 0.98, 95% CI 0.97–0.99 for premenopausal women; aHR 0.95, 95% CI 0.94–0.96 for postmenopausal women), and those who consumed alcohol on 5 days or more a week showed the highest risk of depression (aHR 1.16, 95% CI 1.12–1.21 for premenopausal women and aHR 1.05, 95% CI 1.01–1.09 for postmenopausal women). Compared with non-drinkers, those who consumed four or fewer drinks a day showed a decreased risk of depression (aHR 0.97, 95% CI 0.96–0.98 for premenopausal women; aHR 0.95, 95% CI 0.93–0.96 for postmenopausal women). On the other hand, premenopausal women who consumed more than 14 drinks a day (aHR 1.19, 95% CI 1.07–1.32) and postmenopausal women who had 8–14 drinks a day (aHR 1.06, 95% CI 1.01–1.11) showed the highest risk of depression.

Regarding physical activity, compared with those who had walked 1 day a week, those who had not walked at least 1 day a week showed an increased risk of depression (aHR 1.05, 95% CI 1.03–1.07 for premenopausal women; aHR 1.03, 95% CI 1.02–1.05 for postmenopausal women), while those who had walked 5 days a week showed the least risk of depression (aHR 0.89, 95% CI 0.87–0.91 for premenopausal women; aHR 0.91, 95% CI 0.90–0.93 for postmenopausal women).

## Discussion

In this study, we investigated the association between smoking, alcohol consumption, and physical activity with the risk of depression. The three major findings of the study include: first, smoking increased the risk of depression in a dose-dependent manner, and the increased risk was higher among premenopausal women compared with postmenopausal women; second, while mild alcohol consumption decreased the risk of depression, heavy alcohol consumption increased the risk of depression, and the association between alcohol consumption and depression was stronger among premenopausal than among postmenopausal women; third, regular physical activity decreased the risk of depression in premenopausal and postmenopausal women. Our results suggest that lifestyle risk factors including smoking, heavy alcohol consumption and physical inactivity are associated with increased risk of depression. The findings were consistent with those of previous studies mentioned in the Introduction.

Cardiovascular changes associated with smoking, heavy alcohol consumption and physical inactivity may affect the occurrence of depression. Smoking is associated with adverse lipid and lipoprotein profiles (37, 38), and alcohol consumption and physical inactivity are associated with increase in blood pressure and levels of fasting glucose (39). Adverse cardiovascular changes can affect the brain, causing focal vascular damage and white matter lesions, which can alter neural connectivity (40, 41). In addition, cardiovascular changes can cause brain dysfunction via inflammation and hypoperfusion that affect the development of depression (41–44). Conversely, symptoms of depression (e.g., decreased motivation or interest) may increase cardiovascular risk factors. The reciprocal relationship between smoking, heavy alcohol consumption, physical activity and depression may lead to the significant results of our study.

The association between lifestyle risk factors and depression is known to be stronger in females than in males (29), and estrogen may play an important role in this association. Estrogen decreases the levels of low-density lipoprotein (LDL) cholesterol and fibrinogen, increases high density lipoprotein (HDL) cholesterol and blood flow, and prevents atherosclerotic changes (38, 45–47). A rapid decline in estrogen levels of perimenopausal and postmenopausal women exacerbates cardiovascular adverse effects, which increases the risk of vascular depression (48). In addition, estrogen has neuroprotective effects mediated via modulation of neuroplasticity and inhibition of neurodegeneration (49, 50).

Along with changes resulting from reduced levels of estrogen, age-related changes in the brain such as decreased expression of brain-derived neurotrophic factor (BDNF) (51, 52) and hippocampal atrophy (53) may be important mechanisms that predispose middle-aged women to depression. However, previous studies have reported that physical activity including aerobic exercise may reverse the risk of aging-related depression by promoting BDNF production and increasing hippocampal volume (53, 54). In addition, other mechanisms may explain the association between lifestyle factors and depression. Nicotine is a psychoactive substance that affects the regulation of monoamine neurotransmitters including dopamine (55). Exercise increases the levels of monoamine neurotransmitters and endorphins (56, 57). Further, physical activity may play a role in preventing depression by enhancing physical fitness and increasing self-esteem (58).

One of major findings is that the risk of smoking and heavy alcohol consumption on depression was higher in premenopausal women than in postmenopausal women. Although this study could not explain the underlying mechanism, longer exposure to unhealthy lifestyle factors may increase the risk of depression and the interaction between estrogen and unhealthy lifestyle factors may increase the risk of depression more than the effect of unhealthy lifestyle factors alone. However, the association is not necessarily causation, and temporal association between them should be examined in the future studies including prospective animal models.

This study demonstrates associations between smoking, alcohol consumption, physical activity and depression in middle-aged women based on real-world data. However, this work has several limitations. First, since the information on lifestyle factors is based on a single health questionnaire conducted at a specific time point, the variables do not necessarily reflect long-term habits. Second, menopausal status was identified only once during the health examination and whether or not menopause was attained during the follow-up was not determined. Therefore, it is difficult to conclude that the interaction between hormones and lifestyle factors persisted until the reported outcome or the end of the observation period. Third, the diagnosis of depression was based on patients' medical records and not clinical evaluation. Clinical information about depression including symptom presentation or severity was lacking. Fourth, the use of medications such as psychotropic medications including antipsychotics, mood stabilizers and antidepressants or other medications for physical illnesses was not included in the analyses. Fifth, since this study was conducted using representative data of South Korea, it is difficult to extrapolate the results directly to other populations worldwide. We identified depression among subjects using medical services records under a diagnosis of depressive disorders. However, it is possible that additional subjects experienced depressive symptoms without using medical services. The use of psychiatric services is affected by attitudes or acceptance of psychiatric illnesses. In Asian countries including South Korea, a negative perception or stigma of psychiatric illnesses is prevalent (59–61), which may lead to insufficient use of psychiatric services and under-reporting of depression.

In conclusion, smoking and heavy alcohol consumption were associated with an increased risk of depression, and the risk is higher in premenopausal women than in postmenopausal women. Regular physical activity was associated with a decreased risk of depression both in premenopausal and postmenopausal women. The results of this study are expected support strategies to correct lifestyle risk factors for the prevention of depression in middle-aged women.

## Data Availability Statement

Publicly available datasets were analyzed in this study. This data can be found here: https://nhiss.nhis.or.kr/.

## Ethics Statement

The study involving human subjects was reviewed and approved by the Institutional Review Board of the Samsung Medical Center. Written informed consent for subjects was not required for this study in accordance with the national legislation and the institutional requirements.

## Author Contributions

HK contributed to the search for background literature, to writing the original draft of the manuscript, and to reviewing. JY and KH contributed to formal analysis. HJJ contributed to conceptualization, project administration, and supervision. All authors contributed to writing and editing the manuscript.

## Funding

This study was supported by Healthcare AI Convergence Research and Development Program through the National IT Industry Promotion Agency of Korea (NIPA) funded by the Ministry of Science and ICT (No. S1601-20-1041), and a grant of the Korea Health Technology R&D Project through the Korea Health Industry Development Institute (KHIDI), funded by the Ministry of Health & Welfare, Republic of Korea (HR21C0885). The sponsor had no role in study design, data collection, data analysis, data interpretation, writing of this paper, or the decision to submit this paper for publication.

## Conflict of Interest

DM has received research support from Nordic Naturals and heckel medizintechnik GmbH. He also works with the MGH Clinical Trials Network and Institute (CTNI), which has received research funding from multiple pharmaceutical companies and NIMH. The remaining authors declare that the research was conducted in the absence of any commercial or financial relationships that could be construed as a potential conflict of interest.

## Publisher's Note

All claims expressed in this article are solely those of the authors and do not necessarily represent those of their affiliated organizations, or those of the publisher, the editors and the reviewers. Any product that may be evaluated in this article, or claim that may be made by its manufacturer, is not guaranteed or endorsed by the publisher.

## References

[1] Baldessarini RJ Forte A Selle V Sim K Tondo L Undurraga J. Morbidity in depressive disorders. Psychother Psychosom. (2017) 86:65–72. 10.1159/000448661 28183075

[2] Sadock BJ Sadock VA Ruiz P. Synopsis of Psychiatry—Behavioral Sciences/Clinical Psychiatry. New York, NY: Lippincott Williams and Wilkins (2015).

[3] Kim GE Jo MW Shin YW. Increased prevalence of depression in South Korea from 2002 to 2013. Sci Rep. (2020) 10:16979. 10.1038/s41598-020-74119-4 33046758 PMC7550589

[4] Weissman MM Bland RC Canino GJ Faravelli C Greenwald S Hwu HG. Cross-national epidemiology of major depression and bipolar disorder. JAMA. (1996) 276:293–9. 8656541

[5] Kessler RC McGonagle KA Zhao S Nelson CB Hughes M Eshleman S. Lifetime and 12-month prevalence of DSM-III-R psychiatric disorders in the United States. Results from the National Comorbidity Survey. Arch Gen Psychiatry. (1994) 51:8–19. 10.1001/archpsyc.1994.03950010008002 8279933

[6] Gater R Tansella M Korten A Tiemens BG Mavreas VG Olatawura MO. Sex differences in the prevalence and detection of depressive and anxiety disorders in general health care settings: report from the World Health Organization Collaborative Study on Psychological Problems in General Health Care. Arch Gen Psychiatry. (1998) 55:405–13. 10.1001/archpsyc.55.5.405 9596043

[7] Rai D Zitko P Jones K Lynch J Araya R. Country- and individual-level socioeconomic determinants of depression: multilevel cross-national comparison. Br J Psychiatry. (2013) 202:195–203. 10.1192/bjp.bp.112.112482 23349294

[8] Albert PR. Why is depression more prevalent in women? J Psychiatry Neurosci. (2015) 40:219–21. 10.1503/jpn.150205 26107348 PMC4478054

[9] Hickey M Schoenaker DA Joffe H Mishra GD. Depressive symptoms across the menopause transition: findings from a large population-based cohort study. Menopause. (2016) 23:1287–93. 10.1097/GME.0000000000000712 27552471

[10] Rocca WA Grossardt BR Geda YE Gostout BS Bower JH Maraganore DM. Long-term risk of depressive and anxiety symptoms after early bilateral oophorectomy. Menopause. (2008) 15:1050–9. 10.1097/gme.0b013e318174f155 18724263

[11] Lagunas N Calmarza-Font I Diz-Chaves Y Garcia-Segura LM. Long-term ovariectomy enhances anxiety and depressive-like behaviors in mice submitted to chronic unpredictable stress. Horm Behav. (2010) 58:786–91. 10.1016/j.yhbeh.2010.07.014 20691693

[12] Li LH Wang ZC Yu J Zhang YQ. Ovariectomy results in variable changes in nociception, mood and depression in adult female rats. PLoS One. (2014) 9:e94312. 10.1371/journal.pone.0094312 24710472 PMC3978042

[13] Harlow SD Gass M Hall JE Lobo R Maki P Rebar RW. Executive summary of the Stages of Reproductive Aging Workshop + 10: addressing the unfinished agenda of staging reproductive aging. J Clin Endocrinol Metab. (2012) 97:1159–68. 10.1210/jc.2011-3362 22344196 PMC3319184

[14] Randolph JF. Jr., Zheng H, Sowers MR, Crandall C, Crawford S, Gold EB, et al. Change in follicle-stimulating hormone and estradiol across the menopausal transition: effect of age at the final menstrual period. J Clin Endocrinol Metab. (2011) 96:746–54. 10.1210/jc.2010-1746 21159842 PMC3047231

[15] Park CY Lim JY Park HY. Age at natural menopause in Koreans: secular trends and influences thereon. Menopause. (2018) 25:423–9. 10.1097/GME.0000000000001019 29112598

[16] Beaglehole R Bonita R Horton R Adams C Alleyne G Asaria P. Priority actions for the non-communicable disease crisis. Lancet. (2011) 377:1438–47. 10.1016/S0140-6736(11)60393-0 21474174

[17] Kearns K Dee A Fitzgerald AP Doherty E Perry IJ. Chronic disease burden associated with overweight and obesity in Ireland: the effects of a small BMI reduction at population level. BMC Public Health. (2014) 14:143. 10.1186/1471-2458-14-143 24512151 PMC3929131

[18] Smith U. Smoking elicits the insulin resistance syndrome: new aspects of the harmful effect of smoking. J Intern Med. (1995) 237:435–7. 10.1111/j.1365-2796.1995.tb00867.x 7738482

[19] Office of the Surgeon General, Office on Smoking Health. Reports of the Surgeon General. In: The Health Consequences of Smoking: A Report of the Surgeon General. Atlanta (GA): Centers for Disease Control and Prevention (US) (2004).

[20] Grant BF Hasin DS Chou SP Stinson FS Dawson DA. Nicotine dependence and psychiatric disorders in the United States: results from the national epidemiologic survey on alcohol and related conditions. Arch Gen Psychiatry. (2004) 61:1107–15. 10.1001/archpsyc.61.11.1107 15520358

[21] Dursun SM Kutcher S. Smoking, nicotine and psychiatric disorders: evidence for therapeutic role, controversies and implications for future research. Med Hypotheses. (1999) 52:101–9. 10.1054/mehy.1997.0623 10340289

[22] Nesse RM Berridge KC. Psychoactive drug use in evolutionary perspective. Science. (1997) 278:63–6. 10.1126/science.278.5335.63 9311928

[23] Adan A Prat G Sanchez-Turet M. Effects of nicotine dependence on diurnal variations of subjective activation and mood. Addiction. (2004) 99:1599–607. 10.1111/j.1360-0443.2004.00908.x 15585051

[24] Pasco JA Williams LJ Jacka FN Ng F Henry MJ Nicholson GC. Tobacco smoking as a risk factor for major depressive disorder: population-based study. Br J Psychiatry. (2008) 193:322–6. 10.1192/bjp.bp.107.046706 18827296

[25] Dixit AR Crum RM. Prospective study of depression and the risk of heavy alcohol use in women. Am J Psychiatry. (2000) 157:751–8. 10.1176/appi.ajp.157.5.751 10784468

[26] Graham K Massak A Demers A Rehm J. Does the association between alcohol consumption and depression depend on how they are measured? Alcohol Clin Exp Res. (2007) 31:78–88. 10.1111/j.1530-0277.2006.00274.x 17207105

[27] Hartka E Johnstone B Leino EV Motoyoshi M Temple MT Fillmore KM. meta-analysis of depressive symptomatology and alcohol consumption over time. Br J Addict. (1991) 86:1283–98. 10.1111/j.1360-0443.1991.tb01704.x 1836410

[28] Lipton R. The relationship between alcohol, stress, and depression in Mexican Americans and non-Hispanic whites. Behav Med. (1997) 23:101–11. 10.1080/08964289709596366 9397282

[29] Yue Y Hong L Guo L Gao X Deng J Huang J. Gender differences in the association between cigarette smoking, alcohol consumption and depressive symptoms: a cross-sectional study among Chinese adolescents. Sci Rep. (2015) 5:17959. 10.1038/srep17959 26639938 PMC4671152

[30] Pasco JA Williams LJ Jacka FN Henry MJ Coulson CE Brennan SL. Habitual physical activity and the risk for depressive and anxiety disorders among older men and women. Int Psychogeriatr. (2011) 23:292–8. 10.1017/S1041610210001833 20863424

[31] Strawbridge WJ Deleger S Roberts RE Kaplan GA. Physical activity reduces the risk of subsequent depression for older adults. Am J Epidemiol. (2002) 156:328–34. 10.1093/aje/kwf047 12181102

[32] Azevedo Da Silva M Singh-Manoux A Brunner EJ Kaffashian S Shipley MJ Kivimaki M. Bidirectional association between physical activity and symptoms of anxiety and depression: the Whitehall II study. Eur J Epidemiol. (2012) 27:537–46. 10.1007/s10654-012-9692-8 22623145 PMC4180054

[33] Lee J Lee JS Park SH Shin SA Kim K. Cohort profile: the National Health Insurance Service-National Sample Cohort (NHIS-NSC), South Korea. Int J Epidemiol. (2017) 46:e15. 10.1093/ije/dyv319 26822938

[34] Shin DW Cho B Guallar E. Korean National Health Insurance database. JAMA Intern Med. (2016) 176:138. 10.1001/jamainternmed.2015.7110 26747667

[35] Yoo KY. Cancer control activities in the Republic of Korea. Jpn J Clin Oncol. (2008) 38:327–33. 10.1093/jjco/hyn026 18407932

[36] Cheol Seong S Kim YY Khang YH Heon Park J Kang HJ Lee H. Data resource profile: the National Health Information Database of the National Health Insurance Service in South Korea. Int J Epidemiol. (2017) 46:799–800. 10.1093/ije/dyw253 27794523 PMC5837262

[37] Craig WY Palomaki GE Haddow JE. Cigarette smoking and serum lipid and lipoprotein concentrations: an analysis of published data. BMJ. (1989) 298:784–8. 10.1136/bmj.298.6676.784 2496857 PMC1836079

[38] Grodstein F Stampfer M. The epidemiology of coronary heart disease and estrogen replacement in postmenopausal women. Prog Cardiovasc Dis. (1995) 38:199–210. 10.1016/s0033-0620(95)80012-3 7494882

[39] Godsland IF Leyva F Walton C Worthington M Stevenson JC. Associations of smoking, alcohol and physical activity with risk factors for coronary heart disease and diabetes in the first follow-up cohort of the Heart Disease and Diabetes Risk Indicators in a Screened Cohort study (HDDRISC-1). J Intern Med. (1998) 244:33–41. 10.1046/j.1365-2796.1998.00312.x 9698022

[40] Alexopoulos GS. Frontostriatal and limbic dysfunction in late-life depression. Am J Geriatr Psychiatry. (2002) 10:687–95. 10.1097/00019442-200211000-00007 12427577

[41] Taylor WD Aizenstein HJ Alexopoulos GS. The vascular depression hypothesis: mechanisms linking vascular disease with depression. Mol Psychiatry. (2013) 18:963–74. 10.1038/mp.2013.20 23439482 PMC3674224

[42] de la Torre JC. Cerebral hemodynamics and vascular risk factors: setting the stage for Alzheimer's disease. J Alzheimers Dis. (2012) 32:553–67. 10.3233/JAD-2012-120793 22842871

[43] Dilger RN Johnson RW. Aging, microglial cell priming, and the discordant central inflammatory response to signals from the peripheral immune system. J Leukoc Biol. (2008) 84:932–9. 10.1189/jlb.0208108 18495785 PMC2538600

[44] Godbout JP Johnson RW. Age and neuroinflammation: a lifetime of psychoneuroimmune consequences. Immunol Allergy Clin North Am. (2009) 29:321–37. 10.1016/j.iac.2009.02.007 19389585

[45] Miller VT LaRosa J Barnabei V Kessler C Levin G Smith-Roth A. Effects of estrogen or estrogen/progestin regimens on heart disease risk factors in postmenopausal women. The Postmenopausal Estrogen/Progestin Interventions (PEPI) Trial. The writing group for the PEPI trial. JAMA. (1995) 273:199–208. 7807658

[46] Miller VT Muesing RA LaRosa JC Stoy DB Phillips EA Stillman RJ. Effects of conjugated equine estrogen with and without three different progestogens on lipoproteins, high-density lipoprotein subfractions, and apolipoprotein A-I. Obstet Gynecol. (1991) 77:235–40. 1846437 10.1097/00006250-199102000-00014

[47] Williams JK Adams MR Klopfenstein HS. Estrogen modulates responses of atherosclerotic coronary arteries. Circulation. (1990) 81:1680–7. 10.1161/01.cir.81.5.1680 2331772

[48] Stewart AL Hays RD Wells KB Rogers WH Spritzer KL Greenfield S. Long-term functioning and well-being outcomes associated with physical activity and exercise in patients with chronic conditions in the Medical Outcomes Study. J Clin Epidemiol. (1994) 47:719–30. 10.1016/0895-4356(94)90169-4 7722585

[49] Do Rego JL Seong JY Burel D Leprince J Luu-The V Tsutsui K. Neurosteroid biosynthesis: enzymatic pathways and neuroendocrine regulation by neurotransmitters and neuropeptides. Front Neuroendocrinol. (2009) 30:259–301. 10.1016/j.yfrne.2009.05.006 19505496

[50] Arevalo MA Azcoitia I Garcia-Segura LM. The neuroprotective actions of oestradiol and oestrogen receptors. Nat Rev Neurosci. (2015) 16:17–29. 10.1038/nrn3856 25423896

[51] Douillard-Guilloux G Guilloux JP Lewis DA Sibille E. Anticipated brain molecular aging in major depression. Am J Geriatr Psychiatry. (2013) 21:450–60. 10.1016/j.jagp.2013.01.040 23570888 PMC3615087

[52] Sibille E. Molecular aging of the brain, neuroplasticity, and vulnerability to depression and other brain-related disorders. Dialogues Clin Neurosci. (2013) 15:53–65. 10.31887/DCNS.2013.15.1/esibille 23576889 PMC3622469

[53] Erickson KI Miller DL Roecklein KA. The aging hippocampus: interactions between exercise, depression, and BDNF. Neuroscientist. (2012) 18:82–97. 10.1177/1073858410397054 21531985 PMC3575139

[54] Liu PZ Nusslock R. Exercise-Mediated Neurogenesis in the Hippocampus via BDNF. Front Neurosci. (2018) 12:52. 10.3389/fnins.2018.00052 29467613 PMC5808288

[55] Dani JA De Biasi M. Cellular mechanisms of nicotine addiction. Pharmacol Biochem Behav. (2001) 70:439–46. 10.1016/s0091-3057(01)00652-9 11796143

[56] Phillips WT Kiernan M King AC. The effects of physical activity on physical and psychological health. In: Baum A Revenson TA Singer JE editors. Handbook of Health Psychology. Mahwah, NJ: Erlbaum (2001). p. 627–57.

[57] Thoren P Floras JS Hoffmann P Seals DR. Endorphins and exercise: physiological mechanisms and clinical implications. Med Sci Sports Exerc. (1990) 22:417–28. 2205777

[58] McAuley E Blissmer B Katula J Duncan TE Mihalko SL. Physical activity, self-esteem, and self-efficacy relationships in older adults: a randomized controlled trial. Ann Behav Med. (2000) 22:131–9. 10.1007/BF02895777 10962706

[59] Yilmaz E Okanli A. The effect of internalized stigma on the adherence to treatment in patients with schizophrenia. Arch Psychiatr Nurs. (2015) 29:297–301. 10.1016/j.apnu.2015.05.006 26397432

[60] Henderson C Evans-Lacko S Thornicroft G. Mental illness stigma, help seeking, and public health programs. Am J Public Health. (2013) 103:777–80. 10.2105/AJPH.2012.301056 23488489 PMC3698814

[61] Zhang Z Sun K Jatchavala C Koh J Chia Y Bose J. Overview of stigma against psychiatric illnesses and advancements of anti-stigma activities in six Asian societies. Int J Environ Res Public Health. (2019) 17:280. 10.3390/ijerph17010280 31906068 PMC6981757

