# Supplementary material for: Associations Between Smoking, Alcohol Consumption, Physical Activity and Depression in Middle-Aged Premenopausal and Postmenopausal Women
Source: Front Psychiatry. 2021 Dec 23;12:761761. doi: 10.3389/fpsyt.2021.761761 (PMC8733565; doi:10.3389/fpsyt.2021.761761)
Supplement: Supplementary file 1 [file Data_Sheet_1.docx]

**Supplementary Table 1.** Hazard ratios of lifestyle factors associated with the occurrence of depression among those without diagnosis of bipolar disorder

|  | | Premenopausal women | | Postmenopausal women | | |
| --- | --- | --- | --- | --- | --- | --- |
|  |  | Crude | Model | Crude | Model 1 | Model 2 |
|  |  | Hazard Ratio (95% Confidence Interval) | | | | |
| Smoking Status | |  |  |  |  |  |
|  | Never | 1 (Ref.) | 1 (Ref.) | 1 (Ref.) | 1 (Ref.) | 1 (Ref.) |
|  | Ex-smoker | 1.21 (1.16, 1.25) | 1.27 (1.22, 1.32) | 1.05 (1.01, 1.10) | 1.13 (1.08, 1.17) | 1.12 (1.08, 1.17) |
|  | Current smoker | 1.42 (1.39, 1.46) | 1.39 (1.36, 1.43) | 1.16 (1.14, 1.19) | 1.23 (1.20, 1.26) | 1.23 (1.20, 1.26) |
| Alcohol consumption^a^ | |  |  |  |  |  |
|  | None | 1 (Ref.) | 1 (Ref.) | 1 (Ref.) | 1 (Ref.) | 1 (Ref.) |
|  | Mild | 0.96 (0.95, 0.97) | 0.98 (0.97, 0.99) | 0.90 (0.89, 0.91) | 0.95 (0.94, 0.96) | 0.95 (0.94, 0.96) |
|  | Moderate | 1.21 (1.18, 1.24) | 1.12 (1.09, 1.15) | 0.97 (0.94, 1.00) | 1.02 (0.98, 1.05) | 1.02 (0.99, 1.05) |
|  | Heavy | 1.38 (1.32, 1.43) | 1.21 (1.16, 1.26) | 1.02 (0.97, 1.07) | 1.05 (1.00, 1.10) | 1.05 (1.00, 1.10) |
| Regular physical activity | |  |  |  |  |  |
|  | No | 1 (Ref.) | 1 (Ref.) | 1 (Ref.) | 1 (Ref.) | 1 (Ref.) |
|  | Yes | 0.99 (0.97, 1.00) | 0.96 (0.95, 0.97) | 0.95 (0.95, 0.96) | 0.95 (0.94, 0.96) | 0.95 (0.94, 0.96) |

Model: adjusted for age at menarche, parity, duration of breast feeding, history of oral contraceptive use, age, smoking status, alcohol consumption, regular exercise, body mass index, diabetes mellitus, hypertension, and dyslipidemia

Model 1: adjusted for age at menarche, age at menopause, parity, duration of breast feeding, history of oral contraceptive use, duration of hormone replacement therapy, age, smoking status, alcohol consumption, regular exercise, body mass index, diabetes mellitus, hypertension, and dyslipidemia

Model 2: adjusted for duration of fertility instead of age at menarche and menopause in Model 1

^a^Alcohol consumption: mild = up to 15g (equivalent to 1.5 drinks) a day; moderate = 15g to 30g a day; heavy = more than 30g (equivalent to 3 drinks) a day

**Supplementary Table 2.** Hazard ratios of lifestyle factors associated with the occurrence of depression among those with diagnosis of bipolar disorder

|  | | Premenopausal women | | Postmenopausal women | | |
| --- | --- | --- | --- | --- | --- | --- |
|  |  | Crude | Model | Crude | Model 1 | Model 2 |
|  |  | Hazard Ratio (95% Confidence Interval) | | | | |
| Smoking Status | |  |  |  |  |  |
|  | Never | 1 (Ref.) | 1 (Ref.) | 1 (Ref.) | 1 (Ref.) | 1 (Ref.) |
|  | Ex-smoker | 1.26 (0.85, 1.86) | 1.38 (0.92, 2.05) | 1.50 (0.87, 2.59) | 1.40 (0.80, 2.47) | 1.39 (0.79, 2.45) |
|  | Current smoker | 1.37 (1.08, 1.74) | 1.44 (1.12, 1.86) | 1.34 (0.99, 1.83) | 1.46 (1.06, 2.00) | 1.46 (1.06, 2.00) |
| Alcohol consumption^a^ | |  |  |  |  |  |
|  | None | 1 (Ref.) | 1 (Ref.) | 1 (Ref.) | 1 (Ref.) | 1 (Ref.) |
|  | Mild | 0.96 (0.80, 1.15) | 0.93 (0.77, 1.11) | 0.91 (0.73, 1.13) | 0.89 (0.71, 1.12) | 0.90 (0.72, 1.12) |
|  | Moderate | 1.12 (0.80, 1.58) | 0.96 (0.67, 1.37) | 1.32 (0.63, 2.78) | 1.09 (0.51, 2.35) | 1.10 (0.51, 2.36) |
|  | Heavy | 1.29 (0.74, 2.23) | 1.14 (0.65, 2.01) | 0.70 (0.26, 1.86) | 0.62 (0.23, 1.68) | 0.61 (0.22, 1.64) |
| Regular physical activity | |  |  |  |  |  |
|  | No | 1 (Ref.) | 1 (Ref.) | 1 (Ref.) | 1 (Ref.) | 1 (Ref.) |
|  | Yes | 0.92 (0.78, 1.09) | 0.907(0.76, 1.08) | 0.95 (0.80, 1.13) | 0.92 (0.78, 1.10) | 0.92 (0.78, 1.09) |

Model: adjusted for age at menarche, parity, duration of breast feeding, history of oral contraceptive use, age, smoking status, alcohol consumption, regular exercise, body mass index, diabetes mellitus, hypertension, and dyslipidemia

Model 1: adjusted for age at menarche, age at menopause, parity, duration of breast feeding, history of oral contraceptive use, duration of hormone replacement therapy, age, smoking status, alcohol consumption, regular exercise, body mass index, diabetes mellitus, hypertension, and dyslipidemia

Model 2: adjusted for duration of fertility instead of age at menarche and menopause in Model 1

^a^Alcohol consumption: mild = up to 15g (equivalent to 1.5 drinks) a day; moderate = 15g to 30g a day; heavy = more than 30g (equivalent to 3 drinks) a day
